# Mental health first aid certification's impact on Filipino nurses' ability to increase personal help-seeking behaviors, self-awareness of health changes, and improved self-connection

**DOI:** 10.3934/publichealth.2025064

**Published:** 2025-12-24

**Authors:** Maria Elena Holguin, Brenda Marshall, Faith Atte, Katherine J. Roberts

**Affiliations:** 1 School of Nursing, William Paterson University, Wayne, NJ, USA; 2 School of Nursing, Montclair State University, Montclair, NJ, USA; 3 School of Nursing, Rutgers University, Newark, NJ, USA; 4 Teachers College, Columbia University, New York, NY, USA

**Keywords:** mental health first aid, self-care, nurses-ethnic, help-seeking behavior, barriers to health care

## Abstract

**Background:**

Post COVID-19, almost 50% of nurses reported severe depression, and over 30% moderate to severe anxiety. Nurses from minority racial and ethnic backgrounds, including Filipino nurses, accounted for more than 54% of COVID-related deaths, despite comprising only 24.1% of the nursing workforce. Filipino nurses, making up only 1% of the U.S. population, comprise 4.5 % of the nursing workforce and face significant barriers to seeking mental health services, including stigma, a sense of shame, and adherence to cultural values that view mental illness as unacceptable.

**Aim:**

To assess the impact of Mental Health First Aid (MHFA) certification among Filipino nurses to increase behaviors of help-seeking, self-care, and self-awareness of health changes.

**Method:**

A quantitative, quasi-experimental pre- and post-survey design was employed.

**Results:**

There was a significant increase in personal help-seeking behaviors, self-awareness of health changes, and self-connection among Filipino nurses (*n* = 52) post MHFA certification.

**Conclusions:**

MHFA certification for Filipino nurses successfully increased the nurses' ability to seek help, improved awareness of personal health, and improved self-care behaviors.

## 1. Introduction

Filipino nurses constitute approximately 4% (141,000) of the registered nursing workforce in the United States [1],[2]. One in seven immigrant nurses is Filipino, representing 28% of the nation's foreign-born nurses, the largest group. As such, they are integral to the American healthcare system and have been described as its “invisible heroes”. Notably, 24% of nurses who died from COVID-19-related complications were Filipino [1], underscoring their disproportionate exposure to occupational risks. The conditions contributing to depression, anxiety, and burnout in the general nursing population have been exacerbated since the pandemic and have had an even greater psychological impact on Filipino nurses [3]. Addressing and prioritizing their mental health and well-being is essential not only to promote equity and quality of life for this vital workforce but also to ensure the stability, safety, and sustainability of the American healthcare system.

Prior to Covid-19, the prevalence of moderate, moderately severe, and severe depression in the United States for the general population was 12.6%, 2.1% and 0.7% compared to post Covid-19 prevalence of 14.8%, 7.9%, and 5.1% [4]. One in five Americans (approximately 52.9 million) live with mental health challenges [5], with a 25% rise in anxiety disorder [6] and a significant increase in major depressive disorder [5] since Covid-19. In the general nursing community post Covid-19, almost 50% of nurses have reported severe depression and over 30% moderate to severe anxiety [7]. Nurses from minority racial and ethnic backgrounds accounted for more than 54% of COVID-related deaths, despite comprising only 24.1% of the nursing workforce, as they were often deployed in areas with higher exposure risk [8],[9]. Among nurses of color, 42% cited their COVID experience as a major factor in their emotional distress [10]. Half those nurses considered leaving the profession with the negative mental health sequelae from experiencing trauma at work as their reason [10]. Filipino nurses, while making up only 1% of the U.S. population, comprise 4.5% of the nursing workforce [11]. Given the significant emotional distress experienced by nurses of color, including Filipino nurses who make up a disproportionate percentage of the workforce, the mental health challenges they face are further compounded by the risks of Post-Traumatic Stress Disorder (PTSD), burnout, and suicidal ideation post-COVID.

A correlation between PTSD, chronic fatigue, and burnout on the risk of suicide in health care workers has been identified post-COVID [11]. Suicidal ideation, often called suicidal thoughts or ideas, is a broad term that describes a range of contemplation, wishes, and preoccupation with death and suicide [12]. Results from the World Health Organization (WHO) meta-analysis [6] revealed three variables responsible for the increased likelihood of such ideation in healthcare workers: Severe stress, exhaustion, loneliness, and positive COVID-19 diagnosis. Panchal et al. [13] identified that symptoms of depression and anxiety increased by 41% during the pandemic. From the same research study, other issues impacting well-being included difficulties in sleeping and eating, increased use of illicit substances, and deteriorating medical conditions secondary to worry and stress about the virus [13]. When this stress, a typical response to situational pressure and the demands of everyday life, becomes chronic, it can lead to mental health problems [14]. The impact of this stress has proven to be persistent, with some nurses having chronic psychological ramifications [15]. Mokaya et al. [16] identified the added mental health challenges as a contributing factor for nurses leaving the profession. When nurses experience untreated chronic mental health issues, it can impact their desire to stay in their role as bedside nurses [17].

Martinez et al. [18] identified the barriers to seeking mental health services by Filipinos, which include stigma, a sense of shame, and adherence to Asian values of conformity to norms where mental illness is considered unacceptable. As mental illness is an unacceptable disease, it is believed that individuals should use personal resilience to overcome mental health challenges [18]. According to Chan and Litam [19] Filipinos prefer seeking help from family and close friends rather than professionals such as psychiatrists or counselors, who are viewed as outsiders. The close-knit relationships among Filipinos predispose them to disclose physical and mental health challenges, and they are more open and honest with their group. Filipino cultural context fosters a sense of shame associated with mental health issues, further discouraging individuals from seeking help [20]. Moreover, the phenomenon of cultural mistrust plays a critical role in shaping the attitudes of Filipinos toward mental health services. Despite a high likelihood of endorsing the need for mental health support, there is a notable gap in actual service utilization, attributed to mistrust in the healthcare system and a preference for informal support networks [21],[22]. This mistrust is compounded by the stigma surrounding mental health, which is prevalent not only in the general population but also among healthcare professionals, including nurses [23]. The stigma can lead to self-stigmatization, where individuals internalize negative societal attitudes, further hindering their willingness to seek help [24]. Studies have shown that nurses often experience high levels of stigma, which can lead to burnout and a reluctance to disclose their own mental health struggles [25],[26]. This is exacerbated by the demanding nature of their work, especially in the context of the COVID-19 pandemic, which has heightened stress and mental health challenges among healthcare workers [27].

Mental Health First Aid (MHFA), developed and produced by the National Council for Mental Wellbeing, is an evidence-based public education program educating participants to recognize, understand, and respond to signs of mental health challenges. Originating in Australia, MHFA was brought to the United States in 2008. It was modeled after physical first aid courses, emphasizing the importance of early intervention until professional help can be obtained or the crisis resolves [28],[29]. All trainers are provided with standardized instructor training and certification, after which they can administer the MHFA programs (Adult, Youth, and Teen). The MHFA training includes five major steps: Assessing the risk of suicide or harm, listening non-judgmentally, providing reassurance and information, encouraging appropriate professional support, and promoting self-care strategies [30]. This structured approach empowers individuals to act effectively in situations where someone may be struggling with mental health challenges [31]. Our aim of this study was to assess the impact of MHFA training and certification on Filipino nurses' behaviors of self-care, self -awareness of health changes, and improved self-connection.

## 2. Methods

### 2.1. Research question

What impact does Mental Health First Aid (MHFA) training for Filipino nurses have on self-care, self -awareness of health changes, and improved self-connection?

### 2.2. Design

We employed a quantitative, quasi-experimental pre- and post-survey design to assess stigma-related attitudes among healthcare providers and nurses' self-care practices.

### 2.3. Setting

The MHFA training and all data collection procedures, including the administration of surveys, were conducted online. Participants registered for MHFA through the National Council for Mental Wellbeing's Connect portal. The pre-course videos, asynchronous learning, knowledge pretests and posttests, and access to manuals were provided by the National Council through the Connect portal. The synchronous course was delivered by the instructors via the trainer's Zoom links, and it used the MHFA standard curriculum and slides.

### 2.4. Measures

We utilized two measurement tools:

The Opening Minds Stigma Scale for Health Care Providers (OMS-HC) [32] is a validated scale assessing stigma-related attitudes among healthcare providers. The OMS-HC has demonstrated sensitivity in detecting positive attitudinal changes following anti-stigma interventions [32]. It consists of items rated on a 5-point Likert scale (1 = strongly agree to 5 = strongly disagree), with higher scores indicating greater stigmatization. By examining the attitudes of Filipino nurses, we focused on three factors from this scale: Attitudes of healthcare providers towards people with mental illness, disclosure and help-seeking behaviors, and social distance. Six items were chosen from this measure to assess Filipino nurses' attitudes towards persons with mental illness. This measure was selected to assess the nurses' own feelings related to mental health challenges, which could become a barrier to self-identification of challenges and referral for treatment.

The Self-Care Activities Screening Scale (SASS-14) is a 14-item measure evaluating engagement in self-care behaviors [33]. The SASS-14 utilizes a six-point Likert scale and was developed during the pandemic to measure specific self-care activities. Higher self-care scores indicate higher levels of self-care behaviors and well-being. This scale has been identified as being helpful in addressing the impact of interventions that improve or promote healthy behaviors [33]. Six items from this scale were selected for use in this study, assessing Filipino nurses' self-care before and after the MHFA certification course, enabling a comparison of self-care initiatives before and after the MHFA certification. These questions included: (1) Be alert to changes in my health, (2) do physical activity at least 30 minutes a day, (3) seven to eight hours of sleep, (4) actively participate in the initiatives of my community, (5) be more connected to self, and (6) eating better than I used to.

### 2.5. Sample and recruitment

A purposive convenience sample of Filipino nurses was selected to ensure that participants met, our inclusion criteria. This sampling strategy was appropriate because we sought to explore the experiences and perspectives of a defined professional and cultural subgroup; Filipino nurses actively practicing in the healthcare field at the time of data collection. Following Institutional Review Board (IRB) approval, participants were recruited through multiple sources to enhance accessibility and diversity within the sample. Participants were invited via email announcements distributed through the Philippine Nurses Association, posts on the Philippine Nurses Association of New Jersey (PNANJ) website, social media postings (e.g., Facebook), and messages sent to graduate nursing program mailing lists. The purposive convenience design enabled efficient recruitment of qualified participants from a geographically dispersed population, ensuring that the sample reflected, our focus while maintaining feasibility within the research timeframe.

### 2.6. Inclusion and exclusion criteria

Participants were eligible if they (a) held an active nursing license, (b) were employed in any healthcare setting within the United States, and (c) self-identified as being of Filipino heritage. “Self-identified” was defined as individuals who reported Filipino ancestry, whether through birth in the Philippines or Filipino parentage, and who personally identified as Filipino in cultural or ethnic terms. Exclusion criteria included individuals who were not nurses, did not identify as Filipino, or were residing outside the United States at the time of data collection.

### 2.7. Procedure

*Registering for MHFA*: Two certified MHFA trainers delivered the Adult MHFA certification courses to all nurses who registered for the program. MHFA certification training is a standardized program, similar to certification in Cardio Pulmonary Resuscitation (CPR) courses, in that it follows the established curriculum, which was created by the Council of Mental Well Being. The National Council has a website portal called ‘connect’, on which each participant registers, enabling them to sign on for, and engage in, the asynchronous MHFA learning modules, as well as access the knowledge and satisfaction surveys and certificate of completion (certification in MHFA). Quick Response (QR) codes were provided to all registered participants for the two scales specific to this study, along with informed consent forms. Qualtrics was the platform used to collect this data.

*Participant recruitment* began in February 2022 and continued until the last MHFA certification course was completed in November 2022.

*Survey distribution:* Surveys were distributed electronically via Qualtrics. QR codes and direct links were provided via email to access the study's Qualtrics pre-test and post-test surveys.

*Nurse participation*: Upon acceptance to the MHFA certification course, participants completed the mandatory, standardized two-hour asynchronous pre-course program on the Connect portal and then joined one of three 5-hour standard MHFA synchronous online Zoom MHFA certification courses. All nurses completing the full certification program, regardless of their participation in the study, received free 3-year national MHFA certification. Those who self-identified as Filipino nurses and who agreed to participate in the study had their pre-test survey responses recorded. Three months post-certification, each participant, self-identifying as a Filipino nurse who had agreed to participate in the study, completed the post-test survey.

### 2.8. Data collection and ethical considerations

The Institutional Review Board approval was obtained from William Paterson University (IRB Protocol #2022–340). All participants provided electronic informed consent prior to any data collection. All respondents were informed that their participation in the study was voluntary.

Study withdrawal: Participants were notified in the informed consent that participation was optional, and withdrawing from the study at any time (choosing not to complete the study surveys) would not incur any penalties or impact their certification in MHFA. Surveys that had been submitted, however, could not be removed as the responses were all anonymous and the results aggregated. All participants received an explanation of our purpose and procedure. To maintain participant confidentiality, all responses were collected anonymously, preventing the ability to link individual pre-test and post-test responses.

### 2.9. Data analysis

Data were collected using Qualtrics and imported into the Statistical Package for the Social Sciences (SPSS, Version 29) for statistical analyses. Descriptive statistics, including frequencies and percentages, were used to summarize the demographic characteristics of the sample. Independent sample *t*-tests were conducted to assess changes in Filipino nurses' attitudes and self-care practices from pre-test to post-test. All *t*-tests were two-tailed to enable the detection of differences in either direction. The effect sizes for these comparisons were calculated using Cohen's d.

Data Storage: All survey data were collected through Qualtrics, which provides encrypted, password-protected storage. Following data collection, files were securely transferred to SPSS version 29 for statistical analysis. Access to all electronic data was limited to the principal investigator and designated research team members via password-protected accounts, in compliance with institutional data security protocols.

Missing Data: Because the survey was administered anonymously and data were analyzed in aggregate form, missing responses could not be linked to individual participants. As a result, it was not possible to determine whether missing data reflected participant withdrawal or failure to complete both the pre- and post-surveys. Therefore, missing data were handled using pairwise deletion, enabling analyses to be conducted with all available responses for each variable. Descriptive and inferential statistics were calculated based on valid cases only, and the sample size (*n*) for each analysis was reported accordingly. No data imputation procedures were applied, as anonymity precluded the identification or reconstruction of missing responses.

## 3. Results

### 3.1. Sample characteristics

A total of 52 Filipino nurses participated in the study and received MHFA training and certification. The pre-test was completed by 50 participants, and the post-test was completed by 52 participants. As shown in Table 1, most participants identified as female (78.8%, *n* = 41). The largest age group was nurses over the age of 50 (38.5%, *n* = 20), followed by those aged 41–50 (25.0%, *n* = 13). In terms of marital status, more than half of the participants were married (55.8%, *n* = 29). Most participants were employed in clinical nursing roles (65.4%, *n* = 34), while 26.9% (*n* = 14) worked in administration or management, and 7.7% (*n* = 4) held educator positions (Table 1). There were no missing data for demographic characteristics in the post-test; however, two participants did not complete the pre-test.

**Table 1.** Demographic characteristics of Filipino Nurse participants (*n* = 52).

Characteristic	*n*	%
Gender		
Female	41	78.8
Male	11	21.2
Age		
<31 years	10	19.2
31–40 years	9	17.3
41–50 years	13	25.0
>50 years	20	38.5
Marital Status		
Single	18	34.6
Married	29	55.8
Widowed	4	7.7
Divorced	1	1.9
Nursing Specialty		
Clinical nursing	34	65.4
Administration/management	14	26.9
Educator	4	7.7

### 3.2. Filipino nurses' attitudes toward persons with mental illness

Of the six items measuring attitudes, three showed statistically significant improvements following MHFA certification.

Reluctance to seek help for mental health challenges: Following MHFA certification, participants reported significantly lower reluctance to seek help if experiencing a mental health concern. The difference between pre- and post-certification scores was statistically significant, *t*(82.95) = −8.28, *p* < 0.001, with a large effect size (Cohen's *d* = 1.65). This indicated a strong reduction in internalized stigma related to help-seeking.

Willingness to live near individuals with mental illness: Participants' willingness to live next door to someone with a mental illness significantly increased post-certification, *t*(95.80) = −1.90 and *p* = 0.060. While this result approached significance, it did not meet the conventional threshold (*p* < 0.05) in a two-tailed test and should, therefore, be interpreted with caution. The effect size was small to moderate (Cohen's *d* = 0.38), suggesting limited practical importance despite marginal statistical significance.

Awareness of Personal Health Changes: Participants demonstrated significantly greater awareness of their health post-certification, *t*(52.17) = −3.51 and *p* < 0.001, with a moderate effect size (Cohen's *d* = 0.71). This finding reflects increased mindfulness and health monitoring following the training.

Three items did not show statistically significant change from pre- to post-certification, including comfort helping a person with mental illness, beliefs about individuals with mental illness not trying hard enough to recover, and participation in community initiatives.

### 3.3. Self-care behaviors

Of the five self-care items analyzed, three demonstrated statistically significant improvements following MHFA certification.

Engagement in Physical Activity: Engagement in physical activity improved significantly after certification, *t*(97.79) = −2.11 and *p* = 0.037, with a small to moderate effect size (Cohen's *d* = 0.42). This suggested the course may have encouraged behaviors linked to stress relief and overall well-being.

Sense of Self-Connection**:** There was a statistically significant increase in participants' ability to find moments of self-connection post-certification, *t*(72.29) = −2.97 and *p* = 0.004. The effect size was moderate (Cohen's *d* = 0.59), suggesting that the course may have supported emotional self-awareness and reflection.

Healthy Eating Habits: Participants reported healthier eating habits after the course, such as reducing sugar, salt, and processed food intake. This change was statistically significant, *t*(90.46) = −3.11 and *p* = 0.002, with a moderate effect size (Cohen's *d* = 0.62), pointing to broader health behavior improvements.

Two self-care items did not show statistically significant changes. Participants' sleep duration (7–8 hours per night) demonstrated a trend toward improvement, but the change did not reach statistical significance, *t*(99.88) = −1.84 and *p* = 0.068, with a small to moderate effect size (Cohen's *d* = 0.37), suggesting potential practical relevance. Similarly, participation in community initiatives remained unchanged from pre- to post-certification, *t*(99.97) = 0.14 and *p* = 0.887.

## 4. Discussion

We examined the impact of MHFA certification on Filipino nurses' attitudes toward individuals with mental illness and their engagement in self-care practices. Consistent with research demonstrating MHFA's effectiveness in reducing mental health stigma [28],[30], we found a significant decrease in participants' reluctance to seek help for their own mental health concerns. This shift is particularly important given the cultural context of Filipino nurses, where stigma, shame, and a preference for informal support networks often deter help-seeking [18],[20]. The training appeared to support a critical mindset shift by reinforcing the acceptability and importance of seeking care. Some attitudes, however, remained unchanged after certification. Participants' beliefs about the effort individuals with mental illness put into recovery and their comfort level in helping those with mental illness did not significantly shift post-certification. These beliefs may reflect the nurses' professional specialties in general non-psychiatric nursing and inexperience working with patients with mental illness, thereby making their belief system more resistant to changes.

In addition to improved attitudes toward personal help-seeking, significant increases in self-awareness of health changes and self-connection suggest that MHFA may also enhance emotional regulation and mindfulness. These are two protective factors against burnout and compassion fatigue [25],[27]. Significant positive behavioral changes in physical activity and healthy eating were found and can help buffer against stress and mental health challenges [14]. Two self-care sleep duration and community involvement did not show significant improvement. While sleep patterns trended positively, their lack of statistical significance may be due to systemic workplace factors, such as shift schedules, which MHFA training alone cannot resolve. The absence of change in community engagement may reflect participants' high level of community engagement (76%) prior to the certification.

Performing self-care activities is associated with building self-efficacy, as people who engage with self-care activities have the willingness to promote health and wellbeing [32]. Jiang et al. [34] found that lack of self-care among health service professionals led to compromised quality outcomes and burnout. Self-care helps identify and manage such challenges as stress and interpersonal difficulties.

## 5. Limitations

This study has several limitations that should be acknowledged. The sample size of 52 participants, while meaningful for exploratory purposes, limits statistical power and the ability to detect smaller effect sizes. In addition, the sample was restricted to Filipino nurses residing in a single U.S. state, which may limit the generalizability of findings to the broader population of Filipino nurses nationwide or internationally. All survey responses were collected anonymously and aggregated to protect participant confidentiality, which prevented the ability to conduct within-subject analyses or assess individual-level changes over time. As the survey was anonymous and data were analyzed in aggregate form, missing responses were handled by pairwise deletion. Analyses were conducted using all available data for each variable, and the sample size (n) for each item was reported accordingly. No imputation methods were applied. Given the voluntary nature of participation and the use of a convenience sample, the results may not be generalizable to all Filipino nurses or to nurses from other racial or ethnic backgrounds. These limitations underscore the need for future studies with larger, more diverse samples, culturally adapted MHFA content, and methodologies that enable longitudinal tracking of individual progress.

## 6. Future research

In future studies, researchers should aim to include a more diverse sample of nurses to strengthen the generalizability of findings and examine how cultural, demographic, and professional variables influence perceptions of mental health, stigma, and self-care. Expanding the participant pool would also enable comparative analyses among subgroups of nurses, such as those differentiated by ethnicity, clinical specialty, or years of experience. This level of analysis could clarify how variations in attitudes toward stigma influence nurses' awareness of personal mental health needs and engagement in self-care behaviors. Incorporating qualitative methods, such as structured interviews or focus groups, would enrich future research by providing contextual insight into the quantitative findings, particularly those that are not statistically significant. Mixed-methods approaches could yield a more nuanced understanding of the complex interplay among stigma, self-awareness, and mental well-being in nursing populations. Finally, studies that include culturally tailored adaptations of MHFA and assess its long-term impact on personal well-being and workplace mental health outcomes would provide valuable guidance for supporting and sustaining the nursing workforce.

## 7. Conclusion

The findings from this initial study suggest that MHFA training may serve as an effective intervention to reduce internalized stigma and promote personal wellness behaviors among Filipino nurses, a group shown to experience unique stressors, cultural stigma, and disproportionate risks during and post the COVID-19 pandemic. By fostering greater self-awareness and healthier lifestyle habits, MHFA training addresses critical gaps in mental health education and support within this workforce. While not all measured outcomes showed statistically significant change, the meaningful improvements in help-seeking attitudes and several self-care behaviors highlight the potential of MHFA as a culturally responsive tool to empower nurses to care for themselves and others.

## Use of AI tools declaration

The authors declare that no generative artificial intelligence (AI) tools were used in the writing, data analysis, or preparation of this manuscript. The authors used Grammarly, an assistive language editing tool, solely for grammar, spelling, and clarity improvements in accordance with AIMS Press guidelines.
